# Health Disparity Clusters of Off Label Prescriptions for Glucagon-Like Peptide 1 Receptor Agonists

**DOI:** 10.1016/j.ajmo.2025.100100

**Published:** 2025-04-04

**Authors:** Kateri J. Spinelli, Allison H. Oakes, Shih-Ting Chiu, Mary T. Imboden, Austin Miller, Sanjula Jain, Ty J. Gluckman

**Affiliations:** a Center for Cardiovascular Analytics, Research and Data Science (CARDS), Providence Heart Institute, Providence Research Network, Portland, Ore; b Trilliant Health, Brentwood, Tenn; c The Johns Hopkins University School of Medicine, Baltimore, Md

**Keywords:** Glucagon-like peptide 1 receptor agonists, Health disparities, Off-label prescriptions, Social determinants of health

## Abstract

**Background:**

Off-label prescribing of glucagon-like peptide 1 receptor agonists (GLP-1 RAs) may exacerbate health disparities.

**Methods:**

We performed a retrospective analysis of data from the Trilliant Health national all-payer claims database, US Census Bureau data (race, ethnicity, median household income), and Centers for Disease Control and Prevention social vulnerability index (SVI). Patients with prescriptions for GLP-1 RAs approved for type 2 diabetes mellitus (T2DM) between January 1, 2022, and December 31, 2022 were included. Those without an ICD-10 code for T2DM in their medical claims were considered off-label. Correlations between county-level off-label rates and health disparity variables were examined using visual mapping, geographically weighted regression models, and hierarchical clustering on principle components (HCPC).

**Results:**

A total of 3,688,430 GLP-1 RA prescriptions from 2783 (89%) US counties were included. The median off-label prescribing rate was 37.7% [30.0%-46.3%]. Higher household income was modestly correlated with a higher off-label prescribing rate. HCPC modeling produced seven clusters with distinct geographic locations. The highest off-label prescribing rate (51.6%) occurred in a cluster of counties in Hawaii with high median income ($92,124). The lowest off-label prescribing rate (31.2%) occurred in a cluster of counties that included American Indian Tribal reservation lands, with low median income ($52,437) and high SVI (0.88). Other clusters showed unique patterns of racial and ethnic diversity, income, SVI, and off-label prescribing rates.

**Conclusions:**

We identified distinct populations with varying GLP-1 RA off-label prescribing and known health disparities. These results could inform clinical and market strategies to increase access to GLP-1 RAs in underserved populations.

**Clinical Significance.**

• In this cross-sectional analysis of 3,688,430 prescriptions for diabetes-approved glucagon-like peptide 1 receptor agonists (GLP-1 RA) from a 2022 all-payer US claims dataset, 38% were off-label.
• Machine learning models identified geographically distinct clusters of populations with varying off-label rates and differences in race, ethnicity, median income, and social vulnerability.
• Equitable access to GLP-1 RAs likely requires regional population health strategies to overcome existing practice patterns.

Alt-text: Unlabelled box

## Introduction

Glucagon-like peptide 1 receptor agonists (GLP-1 RAs) represent an important medication class, initially approved for type 2 diabetes mellitus (T2DM) and more recently weight loss. Prescriptions for GLP-1 RAs have increased appreciably in the United States (US) over the last several years.^1^^,^^2^ In addition, wide-spread marketing of the weight loss benefits of these drugs has spurred media attention highlighting differences in use and access between over-resourced and under-resourced populations.^3^, ^4^, ^5^ Previous research studies evaluating patients with T2DM found racial, ethnic, and socioeconomic disparities in prescribing patterns of GLP-1 RAs.^6^^,^^7^ Whether this pattern exists in those taking GLP-1 RAs off-label remains to be determined. We sought to examine off-label prescribing of GLP-1 RAs approved for T2DM across the US through the lens of race, ethnicity, and social determinants of health.

## Methods

We performed a retrospective analysis of the Trilliant Health national all-payer claims database, which includes inpatient, outpatient, and prescription drug claims from all 50 states and the District of Columbia. We analyzed claims aggregated at the county level to identify GLP-1 RA prescriptions filled between January 1, 2022, and December 31, 2022. The following GLP-1 RAs approved for T2DM were included: liraglutide (Victoza), semaglutide (Ozempic, Rybelsus), and tirzepatide (Mounjaro). Patients with at least one GLP-1 RA prescription were reported; those with prescriptions for multiple GLP-1 RAs were only counted once. The on-label prescription rate was defined by the percentage of patients prescribed a GLP-1 RA with one or more related ICD-10 codes for T2DM [E11.XX] present in their medical claims between January 1, 2017, and December 31, 2022. The off-label prescription rate was defined as 1−the on-label rate. In accordance with the National Human Protections Committee Guidelines, this study was exempt from review and informed consent because the data were de-identified.

Population characteristics, including seven race categories (White, Black or African American, Asian, American Indian or Alaska Native, Native Hawaiian or Pacific Islander, other race, or two or more races), ethnicity (Hispanic or non-Hispanic), and median annual household income, were obtained at the county-level using 2022 US Census Bureau American Community Survey 5-year estimates.^8^ An exception to this was Connecticut—since the 2022 census data contained planning regions rather than counties, the 2021 census data was used to obtain county information. Social determinants of health were assessed using the most recent (year 2020) Centers for Disease Control and Prevention (CDC) social vulnerability index (SVI) composite score.^9^ The SVI aggregates 16 census data points on a scale of 0 to 1, with higher numbers indicating increased social vulnerability.

In the claims dataset, county assignment for each patient was based on where the majority of their medical claims were filed, rather than their home address. County-level claims population was calculated as the total number of unique patients within the county in 2022. Thus, the county population could vary between the claims dataset and the census dataset. The following exclusions were applied: counties or territories not included in census data (n = 69), counties with off-label drug use not available (n = 34), counties with low representation in the claims dataset (<10% of the census population [n = 255]), and counties with small census populations (<2500 people) which may cause inflated off-label percentage calculations (n = 93) (Supplementary Figure 1).

### Study aims

The primary aim was to identify correlations between county-level GLP-1 RA off-label prescription rates and health disparity variables (race, ethnicity, median household income, and social vulnerability) within the US. Our hypothesis was that higher rates of off-label prescribing would be present in counties that had higher percentages of White, non-Hispanic residents, higher median household income, and lower social vulnerability.

### Statistical analysis

Categorical data were presented as frequencies (percentages) and numeric data were presented as mean (standard deviation [SD]) or median (interquartile range [IQR]), as appropriate. The off-label prescribing rate was categorized into quartiles and differences in population characteristics between quartiles were analyzed using the Kruskal–Wallis rank sum test.

To explore the spatial variations in the relationships between off-label prescription rates and several independent variables, we first employed geographically weighted regression (GWR) analysis. The dependent variable in this analysis was the off-label prescription rate. The independent variables were the proportions of various races (White, Black or African American, Asian, American Indian or Alaska Native, Native Hawaiian or Pacific Islander, and two or more races), ethnicity (Hispanic or Latino), median household income, and SVI. Further details related to the GWR analysis are included in the Supplementary Methods.

An alternative, unsupervised machine learning approach (hierarchical clustering on principal components [HCPC] analysis) was employed to investigate multidimensional interactions between all included variables and to explore relationships between off-label prescription rates, demographics, and social vulnerability across the US. To identify trends across the 2783 US counties and present interpretable, low-dimensional results, we incorporated 13 population features, including off-label prescription rates, county size, medication claim counts, and health disparity variables (race, ethnicity, household income, and SVI). All original variables were standardized individually, and principal component analysis was used to transform them into independent linear combinations, addressing collinearity while retaining all variables and their explanatory power. This process yielded seven uncorrelated principal components, explaining 92% of the total variance. Next, hierarchical clustering was performed using Ward's criterion on the derived principal components, successfully partitioning the counties into seven clusters based on the variation within the principal components. This clustering approach reduced the 2783 counties into a manageable number of geographically meaningful regions, facilitating summarization and comparison of shared trends within and across clusters. A sensitivity analysis was also conducted using an HCPC model that excluded the off-label prescribing rate.

Maps were created to visualize the geographical distribution of clusters based on the 5-digit Federal Information Processing Standard (FIPS) codes for US states and counties. There were no missing data after exclusion criteria were applied. Significance was considered at *P*-values <.05. All analyses were conducted in R version 4.3.1, using GWmodel package for GWR and FactoMineR package for HCPC. Adobe Illustrator 2022 was used for figure design edits.

## Results

A total of 3,688,430 GLP-1 RA prescriptions from 2783 (89%) US counties were included (Supplementary Figure 1). The prevalence of prescriptions per county varied from 0.15% to 0.85% depending on the drug (Supplementary Table 1). The median on- and off-label prescription rates per county were 62.3% [53.7%-70%] and 37.7% [30.0%-46.3%], respectively (Table 1). Residents of the included counties were 84.6% White, 2.5% Black, 0.7% Asian, 0.3% American Indian or Alaska Native, <0.1% Native Hawaiian or Pacific Islander, 1.5% other races, and 4.6% of two or more races; 4.8% were of Hispanic or Latino ethnicity. The median household income was $61,286 [$53,033-$71,058] and the mean SVI was 0.51 (0.29).

**Table 1:** Population Summary.

Variable	All Participants	GLP-1 RA Off-Label Prescription Rate Quartiles				
		Q1 (<30%)	Q2 (30-38%)	Q3 (38-46%)	Q4 (>46%)	*P*-Value
Number of Counties	2783	705	697	690	691	–
Number of Patients per County	16,548 [5512, 61,639]	12,452 [4188, 35,717]	22,402 [7361, 72,326]	21,203 [6776, 86,938]	13,119 [4058, 55,016]	<.001
On-label Prescriptions, %	62.3 [53.7-70.0]	74.8 [72.0-78.9]	65.9 [64.0-67.9]	58.3 [56.2-60.3]	46.7 [41.1-50.5]	<.001
Off-label Prescriptions, %	37.7 [30.0-46.3]	25.2 [21.1-28.0]	34.1 [32.1-36.0]	41.7 [39.7-43.8]	53.3 [49.5-58.9]	<.001
Race, %*						
White	84.6 [69.4, 92.2]	88.7 [72.0, 93.8]	83.9 [69.2, 91.9]	83.2 [68.1, 91.6]	82.6 [69.2, 90.3]	<.001
Black or African American	2.5 [0.8, 10.6]	1.4 [0.6-5.9]	2.7 [0.9-10.7]	3.4 [0.9-12.5]	3.6 [0.9-13.2]	<.001
Asian	0.7 [0.3, 1.4]	0.6 [0.3, 1.0]	0.7 [0.4, 1.6]	0.8 [0.4, 1.7]	0.7 [0.3, 1.5]	<.001
American Indian or Alaska Native	0.3 [0.1, 0.8]	0.3 [0.1, 0.9]	0.3 [0.1, 0.7]	0.3 [0.1, 0.7]	0.4 [0.2, 0.9]	.006
Native Hawaiian or Pacific Islander	0.0 [0.0, 0.1]	0.0 [0.0, 0.1]	0.0 [0.0, 0.1]	0.0 [0.0, 0.1]	0.0 [0.0, 0.1]	<.001
Other Race	1.5 [0.6, 3.2]	1.1 [0.5, 2.8]	1.5 [0.7, 3.3]	1.6 [0.7, 3.4]	1.6 [0.7, 3.0]	<.001
Two or more Races	4.6 [3.2, 7.0]	4.1 [3.0, 6.1]	4.7 [3.3, 7.4]	4.9 [3.3, 7.4]	4.8 [3.4, 7.1]	<.001
Hispanic or Latino Ethnicity, %*	4.8 [2.6-10.6]	3.9 [2.3-8.6]	4.8 [2.7-11.7]	5.3 [2.7-11.1]	5.4 [2.9-10.7]	<.001
Median Household Income, $*	61,286 [53,033, 71,058]	60,456 [53,318, 68,540]	62,506 [54,903, 71,250]	61,678 [52,970, 73,276]	59,834 [51,529, 73,040]	.004
Social Vulnerability Index	0.5 (0.3)	0.5 (0.3)	0.5 (0.3)	0.5 (0.3)	0.5 (0.3)	.061

⁎ Based on census population. GLP-1 RA = Glucagon-like peptide 1 receptor agonist. Data presented as median [interquartile range] or mean (standard deviation).

**Figure 1:**
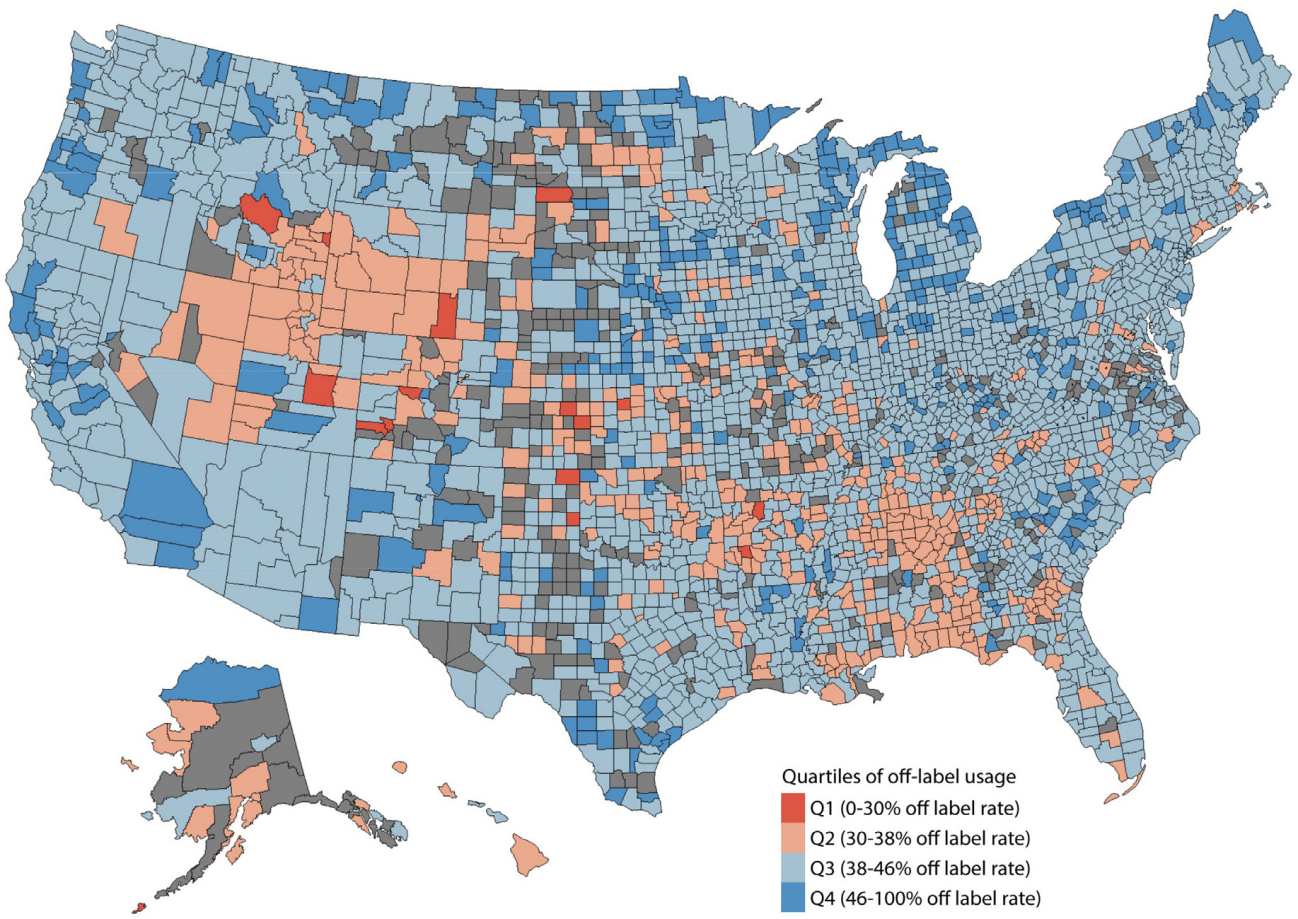
Off-label Prescription Rates for T2DM GLP-1 RA Drugs in US Counties. Legend: Rates were categorized into quartiles for visualization. Counties excluded from the analysis are shown in grey.

Off-label prescription rates were heterogeneous across US counties (Figure 1). The highest quartile of off-label prescribing (>46%) was observed in counties with higher rates of Black residents (3.6% in Q4 vs 1.4% in Q1), residents of other races (1.6% in Q4 vs 1.1% in Q1), and Hispanic or Latino residents (5.4% in Q4 vs 3.9% in Q1) (Table 1). While comparisons between quartiles were statistically significant, differences in absolute values were small. Bivariate mapping of off-label prescription rates and SVI reaffirmed heterogeneity across the US, with few examples of clear regional correlation (Supplementary Figure 2). Based on GWR models, higher household income was the only estimate in the model that showed a positive trend for higher off-label prescribing across the entire US (0.00008 [0.00002, 0.00016]) (Supplementary Table 2). Race, ethnicity, and SVI did not consistently correlate with higher or lower off-label prescription rates across the US. GWR model performance indicated local multicollinearity and low R^2^ values (R-squared = 0.424).

**Figure 2:**
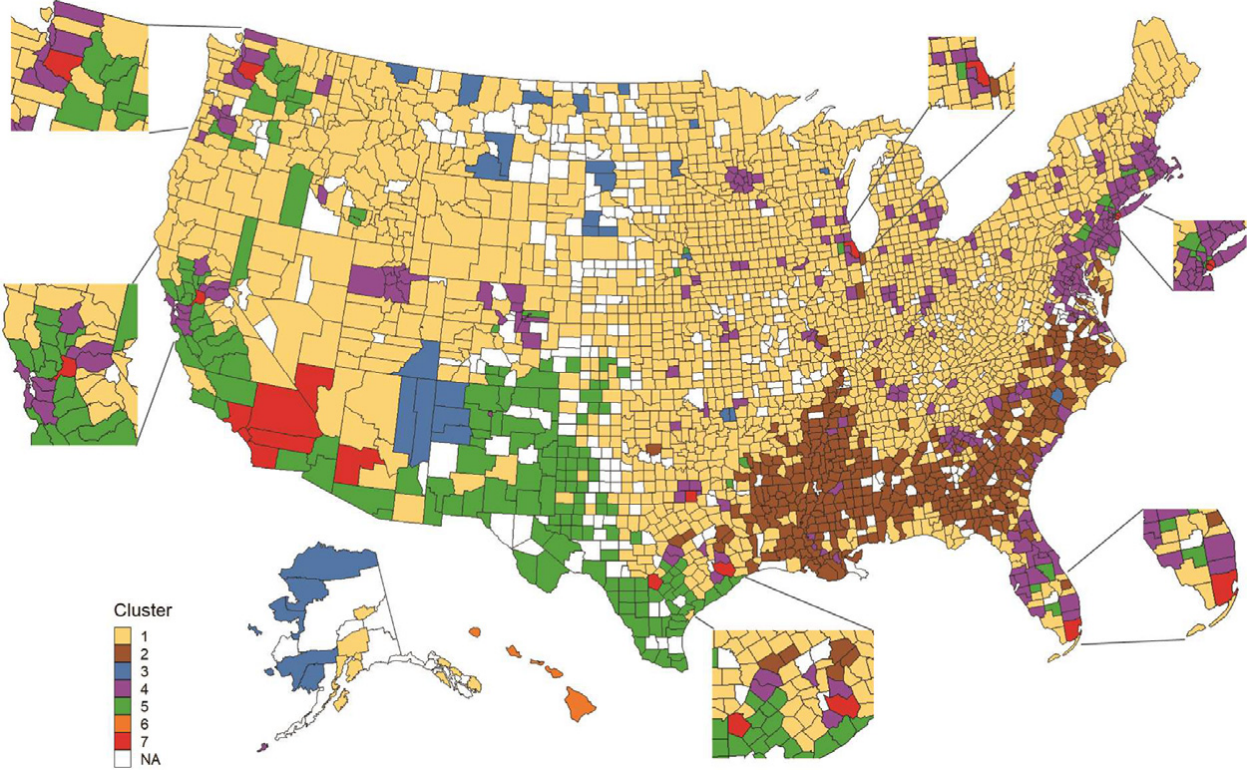
Clusters of US Couties Based on Race, Ethnicity, Median Household Income, Social Vulnerability Index, and Off-Label Prescription Rates for T2DM GLP-1 RA Drugs. Legend: Geographic localization of the seven clusters generated from hierarchical clustering on principle components analysis. Pop outs highlight high-density urban areas where clusters 4, 5, and 7 were found in close proximity. Counties excluded from the analysis are shown in white.

In contrast, the HCPC model accounted for a significantly higher proportion of the total variance (92%) compared to GWR (42%). The HCPC model produced seven clusters with distinct geographic locations across the US (Figure 2). Off-label prescription rates within these clusters varied between 31.2% (cluster 3) and 51.6% (cluster 6) (Table 2). Cluster 1 included a majority of US counties (n = 1876) and as such, had an off-label prescription rate very close to the median (37.0%). Residents of cluster 1 counties were predominantly White (90.2%), with household income close to the national median ($61,564 vs $61,286), and SVI slightly below the mean (0.43 vs 0.51). Cluster 2 was the next largest cluster (n = 403) and included counties that were mostly in the southern US. Residents of cluster 2 counties were 34.4% Black, with the lowest household income ($48,333), the second highest SVI (0.84), and an off-label prescription rate in the third quartile (40.4%).

**Table 2:** Cluster Characteristics.

Variable	Overall	Cluster 1	Cluster 2	Cluster 3	Cluster 4	Cluster 5	Cluster 6	Cluster 7
Number of Counties	2783	1876	403	30	285	168	4	17
Number of Patients per County	16,548 [5512, 61,639]	13,015 [4880, 35,718]	12,099 [5246, 46,520]	3663 [1583, 9955]	265,070 [93,516, 610,417]	21,087 [5917, 137,086]	110,594 [70,671, 250,015]	2,540,776 [1,912,294, 3,239,302]
Off-label Prescriptions, %	37.7 [30.0, 46.3]	37.0 [29.2, 45.6]	40.4 [33.0, 48.7]	31.2 [20.9, 42.9]	42.2 [36.9, 47.9]	32.4 [27.1, 37.6]	51.6 [49.9, 53.6]	35.9 [28.7, 40.3]
Race, %*								
White	84.6 [69.4, 92.2]	90.2 [83.4, 93.8]	57.9 [45.9, 66.2]	32.3 [18.2, 44.6]	73.3 [64.8, 82.1]	65.1 [57.9, 70.2]	30.8 [27.6, 31.5]	49.4 [46.0, 51.2]
Black or African American	2.5 [0.8, 10.6]	1.4 [0.6, 4.0]	34.4 [25.8, 45.7]	0.5 [0.2, 1.1]	9.7 [3.9, 17.2]	2.3 [0.9, 4.9]	0.8 [0.7, 1.2]	9.6 [6.5, 17.7]
Asian	0.7 [0.3, 1.4]	0.6 [0.3, 1.0]	0.6 [0.2, 1.2]	0.6 [0.4, 1.3]	4.0 [2.3, 6.1]	1.0 [0.5, 2.5]	28.9 [26.7, 32.6]	10.2 [6.9, 14.8]
American Indian or Alaska Native	0.3 [0.1, 0.8]	0.3 [0.1, 0.9]	0.2 [0.1, 0.4]	57.4 [42.1, 75.3]	0.3 [0.2, 0.4]	0.8 [0.5, 1.5]	0.3 [0.2, 0.4]	0.7 [0.6, 1.0]
Native Hawaiian or Pacific Islander	0.0 [0.0, 0.1]	0.0 [0.0, 0.1]	0.0 [0.0, 0.1]	0.0 [0.0, 0.1]	0.0 [0.0, 0.1]	0.0 [0.0, 0.2]	10.4 [10.0, 11.0]	0.2 [0.1, 0.3]
Other Race	1.5 [0.6, 3.2]	1.1 [0.5, 2.2]	1.4 [0.6, 2.6]	0.83 [0.2, 2.4]	2.8 [1.8, 4.5]	9.5 [6.6, 13.8]	1.8 [1.5, 2.1]	10.5 [9.0, 13.5]
Two or More Races	4.6 [3.2, 7.0]	4.2 [3.1, 6.1]	3.7 [2.4, 5.2]	4.8 [3.2, 7.1]	6.7 [5.2, 8.3]	16.1 [12.4, 19.6]	27.2 [25.5, 29.1]	13.5 [9.5, 14.9]
Hispanic or Latino Ethnicity, %*	4.8 [2.6, 10.6]	3.9 [2.3, 7.9]	4.2 [2.5, 6.8]	5.1 [2.7, 8.2]	9.0 [5.9, 15.0]	51.2 [40.7, 63.7]	11.7 [11.2, 12.3]	33.9 [26.0, 48.7]
Median Household Income, $*	61,286 [53,033, 71,058]	61,564 [55,044, 69,464]	48,333 [42,508, 55,201]	52,437 [45,748, 61,401]	90,833 [76,912, 104,553]	62,309 [51,722, 71,492]	92,124 [85,211, 96,488]	80,675 [70,789, 84,505]
Social Vulnerability Index	0.51 (0.29)	0.43 (0.25)	0.84 (0.12)	0.88 (0.11)	0.37 (0.24)	0.83 (0.15)	0.50 (0.11)	0.78 (0.14)

⁎ Based on census population. Data presented as median [interquartile range] or mean (standard deviation). *P*-values for comparisons between all clusters are <.001 for every variable, as determined by the Kruskal–Wallis rank sum test.

Cluster 6 had the highest off-label prescription rate (51.6%). It included four counties in Hawaii with residents who were 10.4% Native Hawaiian or Pacific Islander, 28.9% Asian, 27.2% of two or more races, and 11.7% Hispanic or Latino. Residents of cluster 6 counties had the highest household income ($92,124), with SVI at the mean (0.50). In contrast, cluster 3 had the lowest off-label prescription rate (31.2%). This cluster included American Indian Tribal reservation lands,^10^ of which 57.4% of residents were American Indian or Alaska Native. Residents of cluster 3 counties had the second lowest household income ($52,437) and the highest SVI (0.88).

Cluster 7 had the highest number of patients per county (2,540,776). It included Southern California as well as other counties with major US cities (Seattle, WA, Sacramento, CA, Las Vegas, NV, San Antonio, TX, Houston, TX, Dallas, TX, Miami, FL, New York City, NY, and Chicago, IL). Residents of cluster 7 counties were more often Black (9.6%), Asian (10.2%), of two or more races (13.5%), or other races (10.5%), and 33.9% Hispanic or Latino. The median household income was high ($80,675), as was the SVI (0.78). While clusters 4 and 5 were frequently in close proximity to cluster 7, notable differences were observed. Cluster 4 counties had high population densities and included coastal regions in the Northeastern US and Florida, as well as dispersed counties in other US regions. Residents of cluster 4 counties were predominantly White (73.3%) with higher household income ($90,833) and lower SVI (0.37). Cluster 5 included counties in the southwestern US, California, and select counties in the Pacific Northwest. Residents of cluster 5 counties were predominantly of Hispanic or Latino ethnicity (51.2%), and often of two or more races (16.1%) or other races (9.5%). Household income ($62,309) was similar to the median, with a high SVI (0.83). Rates of off-label prescriptions were in the 3^rd^ quartile for cluster 4 (42.2%) and below the median for clusters 5 and 7 (32.4% and 35.9%, respectively).

In a sensitivity analysis that excluded off-label prescription rates, seven clusters were generated, with a geographic distribution similar to that found in the original model (Supplementary Figure 3). While less variability in off-label prescribing was noted between clusters in this analysis (range of 33.4%-51.6%) (Supplementary Table 3), population characteristics were similar to that observed with the primary HCPC model.

## Discussion

Using a national claims-based dataset, we observed a high rate of off-label GLP-1 RA prescriptions in 2022, with notable heterogeneity across US counties and few clear population-level drivers. Use of an unsupervised machine learning model that included race, ethnicity, median household income, social vulnerability, and rates of off-label prescriptions produced seven clusters with unique geographic patterns.

In our analysis, nearly 40% of GLP-1 RA prescriptions were off-label. In another recent study, first time prescriptions for GLP-1 RAs grew from 4% in the first half of 2018 to 24% in the first half of 2023, with off-label prescriptions growing from 9% to 33% over the same time periods.^1^ Prior national studies found lower odds of on-label prescriptions among American Indian or Alaska Native, Asian, Black, and Hispanic or Latino patients with T2DM, and higher odds of on-label prescriptions in regions with higher median household income.^6^^,^^7^ Similarly, in-line with our hypothesis, linear model results in our study found a positive trend for higher off-label prescription rates in regions with higher household income. However, model performance measures revealed that other factors beyond those included in the model were influencing the results. This suggests that a linear model may not be appropriate due to its low explanatory power and potential biases from collinearity and nonlinear trends. Furthermore, while comparisons between quartiles of off-label prescription rates were statistically significant, percentage differences were small and not clearly meaningful (for example, 5% vs 4% Hispanic or Latino ethnicity), with large confidence intervals indicating population heterogeneity within each quartile. In addition, off-label prescription rates were geographically variable, a pattern that is well-illustrated in the map of off-label rates. Geographic variability in prescriptions for other glucose-lowering drugs has been previously described^11^ and likely reflects population heterogeneity within these counties.^12^^,^^13^ Accordingly, there is a need to reexamine these findings with datasets that extend beyond the county level (eg, ZIP code-level, individual level).

Given the suboptimal performance of linear models, we undertook an exploratory unsupervised machine learning cluster analysis. This method examines the multi-dimensional interactions between variables, allowing for identification of distinct spatial patterns and a clearer understanding of the geographic distribution of off-label prescription rates and their associated factors. The seven clusters identified were largely driven by population characteristics rather than off-label prescription rates, as exclusion of off-label rates from the model resulted in predominantly unchanged drivers of cluster formation. This finding is not unexpected, given the wide variability in race, ethnicity, household income, and social vulnerability of the US population.

The cluster analysis did reveal regions with widely varying rates of off-label prescriptions, including higher rates in Hawaii (51.6%) and lower rates in American Indian Tribal counties (31.2%). Other geographically diverse regions of the US were identified that had strong correlations with off-label prescriptions. For example, cluster 7 included counties in high-density urban areas; adjacent counties formed cluster 4 with predominantly White residents, high median household income, low social vulnerability, and the second highest off-label prescription rate of all the clusters (42.2%). Similarly, a higher off-label prescription rate (40.4%) was observed in the southern US—a region with an increased prevalence of overweight and obesity^14^, as well as T2DM.^15^ The southern US region has also been noted to have higher on-label GLP-1 RA prescriptions.^6^ Despite the availability of separate GLP-1 RA formulations approved for weight loss, limited access to these agents may contribute to utilization of GLP-1 RAs that is technically off-label but likely to be clinically meaningful.^16^^,^^17^

Several important factors outside the scope of this analysis likely contributed to the high off-label prescription rates, as well as the heterogeneity of our results. The multitude of favorable effects of GLP-1 RAs have led to their increased use in other patient populations, including those with cardiometabolic disease but without T2DM (eg, prediabetes,^18^^,^^19^ hypertension,^20^ metabolic dysfunction-associated steatohepatitis^21^), cardiovascular disease,^22^, ^23^, ^24^ chronic kidney disease,^25^ and sleep apnea.^26^ Geographic variability in prescribing behavior, prior authorization requirements, payer preference within specific markets, and geographic differences in adoption of newer medical therapies could have affected off-label prescription rates.^11^^,^^27^, ^28^, ^29^ Future research in these areas, as well as the interplay of cost, access, and market availability, is essential.^30^

Our study contributes to the growing body of evidence that GLP-1 RAs have the potential to widen health inequities. Acknowledging that significant clinical benefit has been demonstrated with use of GLP-1 RAs in patients with T2DM, obesity, and cardiovascular disease—three of the top diseases that contribute to morbidity and mortality in the US and globally—the impacts of these diseases disproportionately affect Black and Hispanic patients, as well as communities with lower income and higher social determinants of health.^15^^,^^31^, ^32^, ^33^ Based on this, some groups have called for governments, nonprofits, and global health organizations to adopt a fair allocation framework for distribution of GLP-1 RAs.^34^^,^^35^ In the absence of widespread adoption of such policies, health systems, clinical practices, and individual clinical care team members should be intentional about implementing best practices for diabetes and weight management. Prescription of GLP-1 RAs should be accompanied by a “prescription” for physical activity and healthy eating, as these lifestyle behaviors have been shown to positively impact cardiovascular health and weight. A comprehensive care approach that includes medications, lifestyle behaviors, and considers a patient’s social determinants of health, will lead to the greatest long-term cardiometabolic and weight benefits.^36^^,^^37^ This approach to care should also help to strengthen the clinician-patient relationship and lessen inequities related to access and utilization of services that stem from mistrust in the medical system within Black, Hispanic, American Indian, and other racial and ethnic communities.^38^^,^^39^ The identification of distinct geographic clusters in this study could also spur partnerships between local public health departments, clinical centers, and community health programs, to support communities most in need of GLP-1 Ras.^40^

## Limitations

As with all claims-based datasets, validity is dependent on accurate documentation. Coding errors (eg, presence of T2DM without a corresponding ICD-10 code) may have contributed to patients being misassigned to off-label use. In addition, because county assignment within the Trilliant Health dataset is based on where most medical claims are filed for an individual patient (rather than a patient’s home address), correlation between claims and census data may be more limited. Finally, because this dataset only captures medical encounters and prescriptions that take place within traditional, insurance-based arrangements, medications paid for out-of-pocket or accessed via compounding pharmacies were not included.

## Conclusions

This retrospective analysis of an all-payer US claims-based dataset found high rates of off-label GLP-1 RA prescribing in 2022. Notable geographic variability was observed, and a consistent pattern of off-label prescriptions based on race, ethnicity, household income, or social vulnerability could not be identified. However, seven geographically distinct clusters of populations with known health disparities and variable off-label prescription rates were identified. These findings could help to inform population health strategies focused on equitable access to GLP-1 RAs.

## Data sharing statement

The claims data in this study will not be made available for sharing. The US Census data and the CDC Social Vulnerability Index data are publicly available and can be accessed through the links included in the reference list.

## CRediT authorship contribution statement

**Kateri J. Spinelli:** Writing – review & editing, Writing – original draft, Visualization, Supervision, Software, Resources, Project administration, Methodology, Investigation, Conceptualization. **Allison H. Oakes:** Writing – review & editing, Writing – original draft, Resources, Methodology, Investigation, Data curation, Conceptualization. **Shih-Ting Chiu:** Writing – review & editing, Writing – original draft, Visualization, Validation, Software, Methodology, Formal analysis, Data curation. **Mary T. Imboden:** Writing – review & editing, Validation, Conceptualization. **Austin Miller:** Writing – review & editing, Software, Methodology, Data curation. **Sanjula Jain:** Writing – review & editing, Resources, Conceptualization. **Ty J. Gluckman:** Writing – review & editing, Writing – original draft, Methodology, Investigation, Conceptualization.

## Declaration of competing interest

The authors declare that they have no known competing financial interests or personal relationships that could have appeared to influence the work reported in this paper.

## Appendix Supplementary materials

Image, application 1
