# Supplementary material for: Health Disparity Clusters of Off Label Prescriptions for Glucagon-Like Peptide 1 Receptor Agonists
Source: Am J Med Open. 2025 Apr 4;13:100100. doi: 10.1016/j.ajmo.2025.100100 (PMC12151216; doi:10.1016/j.ajmo.2025.100100)
Supplement: Supplementary file 1 [file mmc1.docx]

Supplemental Materials

Supplemental Methods: Geographically Weighted Regression Analysis

Supplemental Figure 1: Study Inclusion Flow Chart

Supplemental Table 1: Drug Prescriptions Within US Counties

Supplemental Figure 2: Off-label Prescription Rates for T2DM GLP-1 Drugs and Social Vulnerability Index

Supplemental Table 2: Global and Geographically Weighted Regression Analyses

Supplemental Figure 3: Clusters of US Couties Based on Race, Ethnicity, Median Household Income, and Social Vulnerability Index

Supplemental Table 3: Cluster Characteristics for HCPC Model Excluding Off Label Prescription Rates

Supplemental References

**Supplemental Methods:** **Geographically Weighted Regression Analysis**

Geographically weighted regression (GWR) analysis was used to explore the spatial variations in the relationships between off-label prescribing rates and the following independent variables: the proportions of various races (White, Black or African American, Asian, American Indian or Alaska Native, Native Hawaiian or Pacific Islander, and two or more races), ethnicity (Hispanic or Latino), median household income, and social vulnerability index (SVI). We used a bi-square kernel function with an adaptive bandwidth to account for spatial heterogeneity. The adaptive bandwidth was set to include the nearest 213 neighbors for each location, ensuring that the local model for each observation was calibrated using data from its nearest neighbors. The Euclidean distance metric was used to measure the spatial distances between observations. The GWR model was calibrated using the same locations as the observations, allowing for the estimation of local regression coefficients for each independent variable at each observation point.

Regression results from the global model and locally varying models are presented in Supplement Table 3. Model performance was compared using the coefficient of determination (R^2^) and corrected Akaike Information Criterion (AICc). The AICc for the global model and GWR was 22034.16 and 21217.47, respectively, indicating evidence of significant non-stationarity. The GWR model demonstrated improved explanatory power (R^2^ = 0.424) compared to the global model (R^2^ = 0.0256), indicating that local factors significantly influence off-label prescription rates. The GWR model provided a detailed understanding of the spatial variations in the relationships between the off-label prescribing rates and the independent variables. The summary of GWR coefficient estimates included the median [first quartile, third quartile], with wide ranges that highlight the spatial variability in these relationships. By using GWR, we were able to capture the local effects of demographic and socioeconomic factors on off-label prescription rates, which varied significantly across different geographic regions. This method provided a more nuanced understanding of the spatial dynamics influencing off-label prescription rates, emphasizing the importance of considering geographic context in such analyses.

While GWR is a valuable method for investigating spatial non-stationarity in regression models, it has notable limitations that influenced our decision to utilize cluster analysis for our final analysis. One significant issue with GWR is its susceptibility to local multicollinearity. Given that predictor values often cluster spatially, GWR models can suffer from high correlations between predictors in localized areas, leading to biased parameter estimates^1^. Furthermore, edge effects can pose substantial challenges in GWR models. These effects, prevalent in most spatial models, result in misleading outcomes for observations at the dataset's periphery^2^. For instance, in metropolitan areas, edge effects artificially limit the neighborhoods considered, omitting adjacent rural tracts and potentially skewing results for boundary observations.

Cluster analysis offers a robust alternative for discerning geographical trends across the US by grouping regions with similar characteristics without the complications of local multicollinearity and edge. Unlike GWR, which is primarily exploratory, hierarchical clustering on principle components (HCPC) provides definitive groupings that are more interpretable and actionable for policy-making and targeted interventions. Therefore, despite the exploratory insights GWR offers, the clarity and robustness of cluster analysis made it a more suitable choice for our study’s final analysis, facilitating a comprehensive understanding of spatial trends that could inform region-specific strategies.

**Supplemental Figure 1: Study Inclusion Flow Chart**

Legend: *2021 census data was used for Connecticut to align with counties instead of planning regions. GLP-1 RA = glucagon-like peptide-1 receptor agonist, GU = Guam, MP = Northern Mariana Islands, PR = Puerto Rico, SVI = Social Vulnerability Index, T2DM = type 2 diabetes mellitus, VI = Virgin Islands


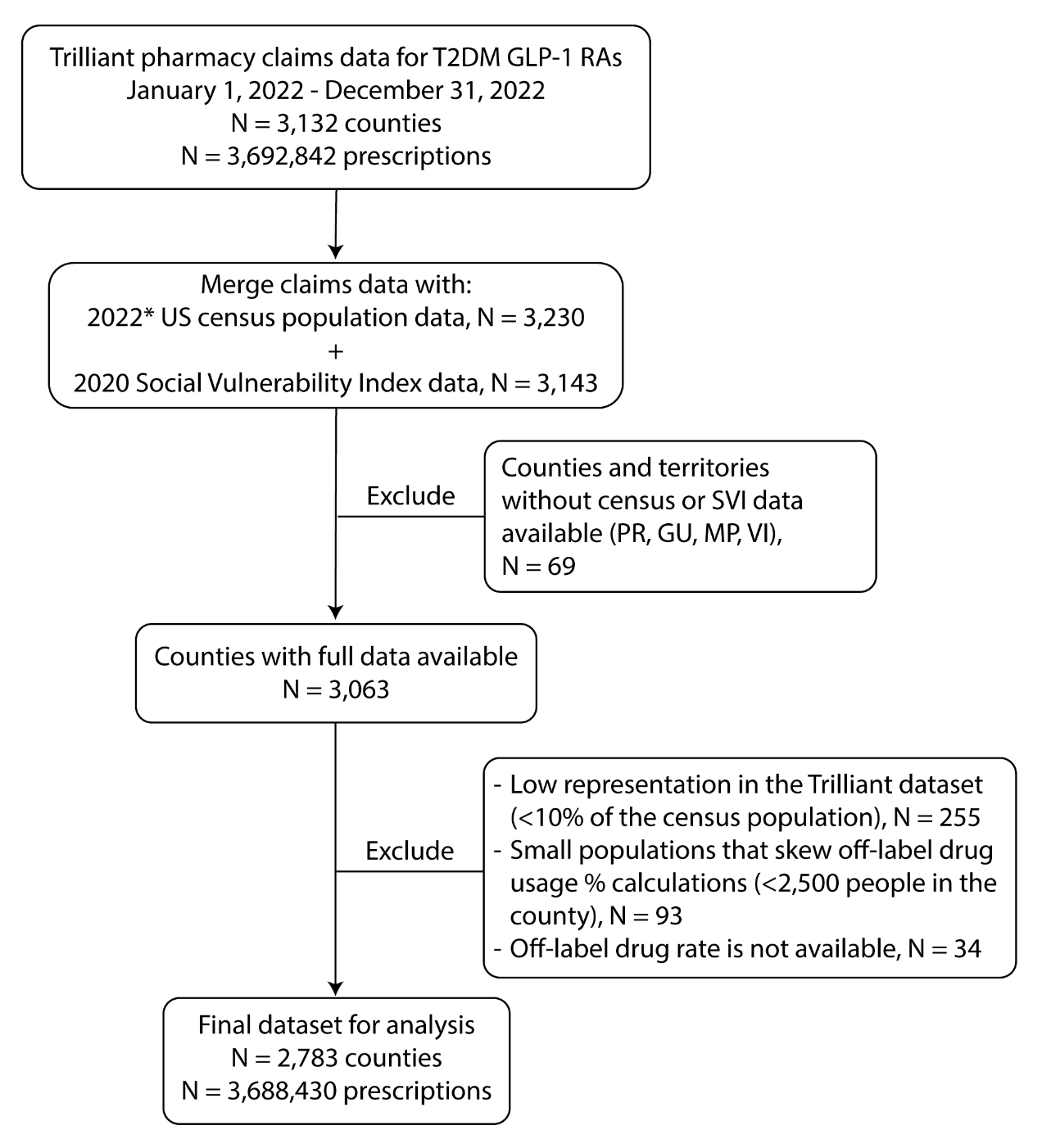


**Supplemental Table 1: Drug Prescriptions Within US Counties**

| **Drug** | **Prevalence*** |
| --- | --- |
| Mounjaro | 0.22% [0.11, 0.42] |
| Ozempic | 0.85% [0.55, 1.18] |
| Victoza | 0.17% [0.11, 0.26] |
| Rybelsus | 0.15% [0.10, 0.23] |

*Based on Trilliant population of each county

Data presented as median [interquartile range].

**Supplemental Figure 2: Off-label Prescription Rates for T2DM GLP-1 RAs and Social Vulnerability Index**

Legend: Bivariate choropleth map showing quartiles of county off-label prescription rates in red gradient, quartiles of social vulnerability index in blue gradient, and the combination of these two variables in mixed red/blue gradient. Counties excluded from the analysis are shown in white. GLP-1 RA = glucagon-like peptide-1 receptor agonist, SVI = Social Vulnerability Index, T2DM = type 2 diabetes mellitus


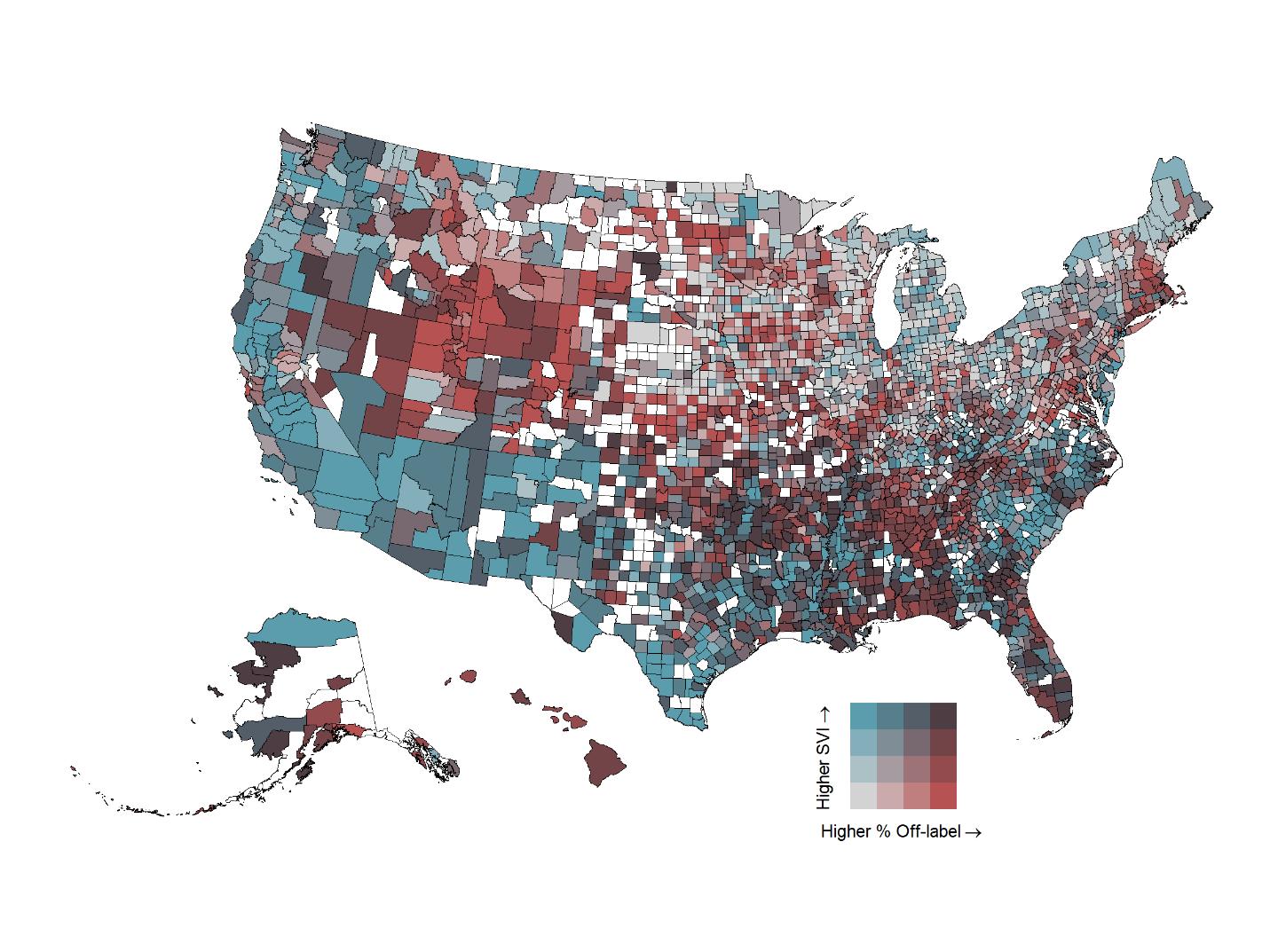


**Supplemental Table 2: Global and Geographically Weighted Regression Analyses**

| **Effect** | **Global Regression** | |  | **Geographically Weighted Regression** |
| --- | --- | --- | --- | --- |
|  | **Estimates** | **p-value** |  | **Estimates** |
| Intercept | 47.58 ± 10.58 | < 0.001 |  | 54.75 [-20.13, 127.44] |
| White | -0.19 ± 0.10 | 0.063 |  | -0.17 [-0.89, 0.54] |
| Black or African American | -0.19 ± 0.10 | 0.063 |  | -0.14 [-0.86, 0.66] |
| Asian | -0.37 ± 0.16 | 0.020 |  | 0.31 [-0.54, 0.98] |
| American Indian or Alaska Native | -0.22 ± 0.11 | 0.045 |  | -0.03 [-1.20, 1.01] |
| Native Hawaiian or Pacific Islander | 1.64 ± 0.63 | 0.009 |  | 1.87 [-2.29, 6.85] |
| Two or More Races | 0.01 ± 0.12 | 0.945 |  | -0.12 [-0.93, 0.87] |
| Hispanic or Latino Ethnicity | -0.20 ± 0.05 | < 0.001 |  | -0.18 [-0.57, 0.22] |
| Median Household Income | 0.00011 ± 0.00002 | < 0.001 |  | 0.00008 [0.00002, 0.00016] |
| Social Vulnerability Index | 6.21 ± 1.54 | < 0.001 |  | -1.50 [-5.81, 3.70] |

Data presented as mean ± standard error or median [interquartile range].

**Supplemental Figure 3: Clusters of US Couties Based on Race, Ethnicity, Median Household Income, and Social Vulnerability Index**

Legend: Geographic localization of the 7 clusters generated from hierarchical clustering on principle components analysis, excluding off-label prescription rates in the model. Counties excluded from the analysis are shown in white.


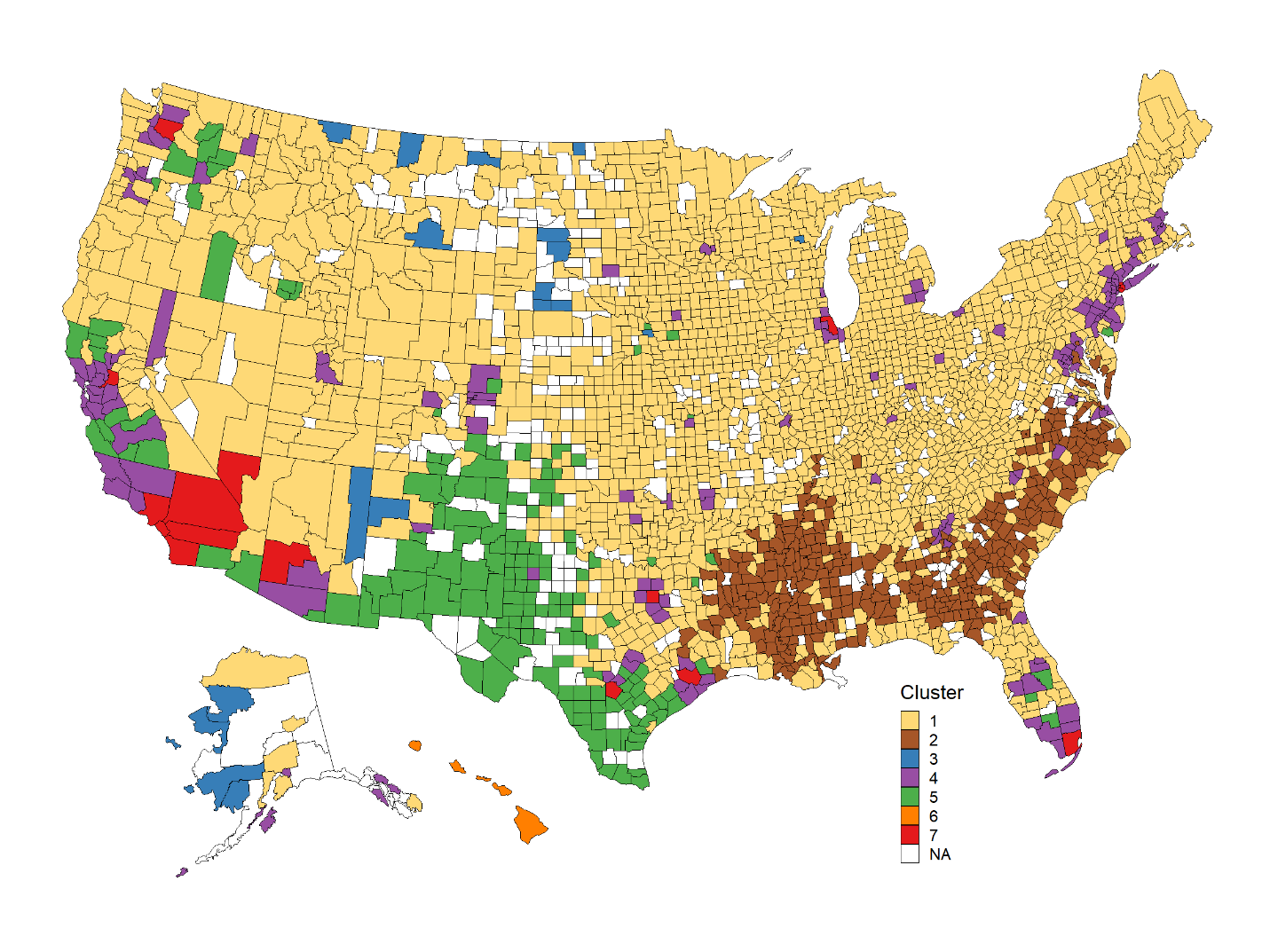


**Supplemental Table 3: Cluster Characteristics for HCPC Model Excluding Off Label Prescription Rates**

| **Variable** | **Overall** | **Cluster 1** | **Cluster 2** | **Cluster 3** | **Cluster 4** | **Cluster 5** | **Cluster 6** | **Cluster 7** |
| --- | --- | --- | --- | --- | --- | --- | --- | --- |
| Number of Counties | 2,783 | 1,876 | 403 | 30 | 285 | 168 | 4 | 17 |
| Number of Patients per County | 16,548  [5,512, 61,639] | 15,461  [5,334, 46,948] | 11,068  [5,071, 39,899] | 2,259  [1,246, 3,820] | 452,424  [215,064, 832,091] | 11,832  [4,545, 34,178] | 110,594  [70,671, 250,015] | 2,372,385  [1,897,215, 3,166,187] |
| Off-label Prescriptions, % | 37.7 [30.0, 46.3] | 37.5 [29.8, 46.2] | 38.2 [31.2, 47.3] | 36.2 [17.2, 55.8] | 41.3 [35.6, 48.0] | 33.4 [27.3, 40.0] | 51.6 [49.9, 53.6] | 35.0 [27.7, 40.2] |
| Race,* % |  |  |  |  |  |  |  |  |
| White | 84.6 [69.4, 92.2] | 89.5 [82.3, 93.5] | 56.8 [44.7, 63.7] | 19.6 [12.4, 32.2] | 64.7 [56.4, 70.8] | 67.6 [59.5, 73.6] | 30.8 [27.6, 31.5] | 48.1 [45.1, 51.0] |
| Black or African American | 2.5 [0.8, 10.6] | 1.6 [0.6, 4.9] | 36.4 [28.7, 47.8] | 0.3 [0.2, 0.9] | 9.2 [3.8, 18.5] | 2.0 [0.9, 4.5] | 0.8 [0.7, 1.2] | 10.8 [6.8, 18.6] |
| Asian | 0.7 [0.3, 1.4] | 0.6 [0.3, 1.2] | 0.5 [0.2, 1.1] | 0.7 [0.3, 1.3] | 5.7 [3.6, 9.2] | 0.7 [0.4, 1.3] | 28.9 [26.7, 32.6] | 9.0 [6.7, 14.2] |
| American Indian or Alaska Native | 0.3 [0.1, 0.8] | 0.3 [0.1, 0.7] | 0.2 [0.1, 0.4] | 72.0 [63.5, 77.3] | 0.4 [0.2, 0.8] | 0.8 [0.4, 1.7] | 0.3 [0.2, 0.4] | 0.7 [0.6, 1.0] |
| Native Hawaiian or Pacific Islander | 0.0 [0.0, 0.1] | 0.0 [0.0, 0.1] | 0.0 [0.0, 0.1] | 0.0 [0.0, 0.1] | 0.1 [0.0, 0.2] | 0.0 [0.0, 0.1] | 10.4 [10.0, 11.0] | 0.2 [0.1, 0.3] |
| Other Race | 1.5 [0.6, 3.2] | 1.2 [0.6, 2.3] | 1.4 [0.6, 2.8] | 0.3 [0.2, 1.0] | 5.8 [3.3, 8.5] | 8.3 [5.6, 12.7] | 1.8 [1.5, 2.1] | 11.3 [9.3, 15.1] |
| Two or More Races | 4.6 [3.2, 7.0] | 4.4 [3.2, 6.1] | 3.4 [2.3, 5.0] | 4.0 [2.9, 5.5] | 9.4 [7.0, 11.9] | 16.1 [12.5, 19.8] | 27.2 [25.5, 29.1] | 13.4 [9.9, 14.9] |
| Hispanic or Latino Ethnicity*, % | 4.8 [2.6, 10.6] | 4.1 [2.4, 7.8] | 4.0 [2.5, 6.7] | 3.6 [2.2, 6.8] | 18.7 [10.6, 26.2] | 51.2 [40.7, 64.4] | 11.7 [11.2, 12.3] | 34.2 [26.5, 50.2] |
| Median Household Income*, $ | 61,286  [53,033, 71,058] | 62,356  [55,245, 71,075] | 48,071  [42,292, 55,277] | 53,135  [44,227, 63,143] | 90,063  [75,026, 104,184] | 57,634  [50,721, 66,713] | 92,124  [85,211, 96,488] | 79,490  [70,746, 84,381] |
| Social Vulnerability Index | 0.51 (0.29) | 0.42 (0.25) | 0.85 (0.12) | 0.88 (0.09) | 0.54 (0.23) | 0.83 (0.15) | 0.50 (0.11) | 0.80 (0.15) |

*Based on census population

Data presented as median [interquartile range] or mean (standard deviation). P-values for each variable between all clusters are <0.001, as determined by the Kruskal-Wallis rank sum test.

**Supplemental References**

1. Brunsdon C, Fotheringham AS, Charlton M. Some Notes on Parametric Significance Tests for Geographically Weighted Regression. *Journal of Regional Science*. 1999;39(3):497-524. doi:10.1111/0022-4146.00146

2. Fotheringham AS, Brunsdon C, Charlton M. *Geographically Weighted Regression: The Analysis of Spatially Varying Relationships*. Wiley; 2002.
